# A survey to assess awareness and opinion of initiatives and recommendations on low-value diagnostic practices

**DOI:** 10.1186/s12913-020-05286-3

**Published:** 2020-06-05

**Authors:** Xavier Bonfill, Karla Salas-Gama, Carolina Requeijo, Angela Merchán-Galvis, Antonio Sánchez, Elena Medarde, M. Jesús Quintana, the DianaHealth Study Group, Dimelza Osorio, Soledad Romea, Francisco Baigorri, Agustín Urrútia, Josep Lluis Vega, Pedro Armario, Matteo Fabbi, Anna Carol Pérez Segarra, Xavier Martret, Miquel Vila, Marta Banqué, Yolima Cossio, Javier Zamora, Jesús López Alcalde, Alfonso Muriel, José Ignacio Emparanza, Iratxe Urreta, José Ignacio Pijoan, Amaia Martínez Galarza, Agustín Gómez de la Cámara, Ana Royuela, Blanca Lumbreras, Pere Plaja, Antoni Peris, Carlos Brotons, Montserrat Ureña, Joan Fernández Náger, Xavier Bonfill, Karla Salas-Gama, Carolina Requeijo, Angela Merchán-Galvis, Antonio Sánchez, Elena Medarde, M Jesús Quintana

**Affiliations:** 1 Centro Cochrane Iberoamericano. Servicio de Epidemiología Clínica y Salud Pública, Hospital de Sant Pau, IIBSant Pau, Barcelona, Spain; 2 CIBERESP, Madrid, Spain; 3 Universitat Autònoma de Barcelona, Barcelona, Spain; 4 Universidad del Cauca, Popayán, Colombia; 5 Consorci Sanitari de Terrassa, Barcelona, Spain

**Keywords:** Appropriateness, Low-value care, DianaHealth, Survey

## Abstract

**Background:**

The need to reduce healthcare practices that provide no value has led to the development of initiatives that generate and publish recommendations to improve the appropriateness of clinical practice by identifying potentially inappropriate services, making recommendations, and proposing improvements. DianaHealth (www.dianahealth.com) identifies, classifies, and publishes recommendations from numerous scientific societies. The purpose of this study was to determine the awareness and perceived usefulness and applicability of published recommendations on low-value diagnostic measures, as judged by physicians who are recognised clinical leaders in their respective centres.

**Methods:**

We designed a questionnaire on the diagnostic recommendations considered relevant for each medical specialty and made it available, until September 2016, on DianaHealth. The survey was administered online to clinical leaders from 25 Spanish healthcare centres (hospitals and primary care centres).

**Results:**

A total of 413 (40.0%) physicians from 34 different specialties participated. The participation rate varied between centres (range 21.1%-100.0%) and specialties (range 12.5%-78.9%). *Do Not Do* (57.1%) was the most widely-known initiative. Most participants (82.6%; IQR 77.9%-94.9%) stated that they knew at least one of the 12 initiatives that identify non-recommended practices, and on average they were aware of four initiatives (range 1-12). The initiatives were perceived useful by 82.4% (IQR 73.3%-90.4%), and perceived applicable by 75.6% (IQR 67.4%-86.8%). A total of 531 recommendations were assessed. Sixty-three percent (IQR 53.6%-77.5%) of participants reported they were aware of the recommendations for their corresponding specialty. A total of 84.5% (IQR 75.0%-90.0%) stated they agreed with the recommendations and 84.5% (IQR 75.0%-90.0%) considered them useful.

Among those who agreed with their respective recommendations, a median of 51.5% (IQR 41.4%-60.9%) perceived the guidelines as being fully implemented, 40.1% (IQR 31.9%-46.8%) considered them partially implemented, and 7.1% (IQR 3.7%-12.9%), not implemented.

**Conclusions:**

Clinical leaders’ awareness of initiatives that generate and publish recommendations to improve clinical appropriateness remains low, although they did consider them useful. In general, participants were familiar with their speciality-specific diagnostic recommendations, agreed with them, and perceived them as useful and implemented in their centres. More needs to be done to raise awareness among professionals who do not know of or apply these recommendations.

## Background

Currently, there is a need to identify and reduce clinical practices of questionable value. Around 20% to 25% of clinical practices have been reported to provide no benefit to the patient and, therefore, present unnecessary risks, with an economic impact that could even exceed 20% of total healthcare costs [1].

Nowadays, optimising health care services is a worldwide major interest, and several recently-published studies have provided a reference framework for appropriate health care [2–5]. Likewise, multiple national and international initiatives that generate and publish recommendations have been developed to improve the appropriateness of care and clinical practice, by identifying potentially inappropriate services, making recommendations, and proposing actions and clinical alternatives, when available, to reduce inappropriate use [6–20].

Most initiatives are conducted by groups of scientific societies who gather clinical recommendations [21]. *Choosing Wisely* [7] is one such example that is active in several countries [12–20]. Other initiatives include a) collections of articles identifying low-value health care practices (e.g. the *Less is More* series in the Journal of the American Medical Association, JAMA [10]); b) ‘do not do’ recommendations (e.g. the *Do Not Do* recommendations from the National Institute for Health and Care Excellence, NICE [6]); c) appropriateness criteria such as those by the American College of Radiology [15]; and d) local projects to improve clinical practice (e.g. ESSENCIAL [9] by the Agency for Health Quality and Assessment of Catalonia (AQuAS), and the MAPAC initiative (in English, Improvement of Appropriateness in Clinical Practice and Healthcare, from the Spanish *Mejora de la Adecuación de la Práctica Asistencial y Clínica*) [21]. In order to overcome the scattered fragmentation of recommendations generated by such initiatives, DianaHealth (www.dianahealth.com) identifies, classifies and publishes them to help clinicians identify the low-value clinical interventions in their particular field [21].

Few studies have been conducted to determine health professionals’ awareness and opinion of the various published recommendations on low-value clinical practices. A study among primary care physicians was conducted in the United States regarding the Choosing Wisely initiative [22]. It found that 66% of participants were aware of the initiative and 97% of those who were acquainted with it considered it an appropriate source of information on low-value tests and interventions. A similar study was developed in Spain at a tertiary hospital regarding the “Do Not Do” recommendations and it found that 90% of the directors of clinical units agreed with the recommendations [23].

The objective of the current study was to determine the awareness of the above-mentioned initiatives and perceived usefulness and applicability in clinical practice of its recommendations on diagnostic practices, in physicians from different healthcare centres in Spain.

## Methods

We conducted a multi-centre, cross-sectional study co-ordinated by the Service of Clinical Epidemiology and Public Health at the Hospital de Sant Pau in Barcelona, and the *Comisión de Mejora de la Práctica Clínica* (Committee for Improving Clinical Practice) of the *Consorci Sanitari de Terrassa* (Barcelona).

From April to September 2017, we conducted a survey aimed at clinical leaders (directors, heads of departments, and heads of clinical units or areas) from hospitals and primary care centres in Spain that had previously communicated an explicit interest to participate in the study. We invited only those professionals whose specialties included on the survey. We sent an email to participate on the study which included a link to the anonymous online questionnaire.

### Recommendation selection

We selected recommendations based on the following criteria:

- Inclusion criteria: recommendations on low-value diagnostic tests, published on DianaHealth from inception (2014) to September 10th, 2016.
- Exclusion criteria: recommendations on non-diagnostic interventions, high-value recommendations, assessments with no explicit recommendation, recommendations with no specialty assigned, or nursing recommendations.

DianaHealth allows users to select recommendations by specialty, so we generated a list for each one. When a recommendation could be classified to more than one specialty, we included it in both lists. We excluded specialties with six or fewer low-value diagnostic recommendations. As a result, 34 specialties were selected.

Between 7 and 25 recommendations were selected for each speciality based on their clinical relevance, general interest, potential clinical impact, and applicability.

### Questionnaire development

The questionnaire consisted of two parts (Additional file 1): the first contained general questions on different initiatives that generate and publish recommendations and the second contained specific questions related to each of the selected recommendations. The responses to the questions about awareness, agreement, usefulness, and applicability were classified as disagreement (“totally disagree” and “disagree”), neither agree nor disagree, and agreement (“agree" and "totally agree”).

The survey was designed and carried out in accordance with the Checklist for Reporting Results of Internet E-Surveys (CHERRIES) [24]. Before starting, we tested the survey for usability and technical functionality. Each physician was invited to answer both sections of the questionnaire through the electronic platform Clinapsis®. The survey was open for three months, from May to July 2017. The time needed to complete the survey was around 10 to 20 minutes, depending on the number of recommendations for each speciality.

We conducted a descriptive data analysis for all the collected variables, expressed as percentage of respondents, using the software Stata version 14.0. Participation rate was calculated considering those clinical leaders that answered the survey divided by the target population (clinical leaders who were invited to answer the survey).

The study was approved by the Clinical Research Ethics Committee of both co-ordinating centres. No informed consent was required since the questionnaire was completed anonymously. Our online survey did not require a signed consent, because it was completely anonymous since it did not have any information that could be used to glean the identity of the participants. Nevertheless, the protocol of the study was presented to two ethics committees and they considered not necessary to be review by an ethical research committee since it was not a biomedical research.

## Results

Initially, 25 centres agreed to participate in the study, although 7 were excluded from the analysis: 2 because they did not reach the minimal participation rate (< 5%) and 5 because finally they decided not to take part of the survey. The majority of participating centres were hospitals (15), but three primary centres also participated (3). The target population consisted of 1,030 physicians, 413 of whom completed the survey (participation rate: 40.1%). Six centres had a participation rate exceeding 50% (Table 1). If we would have considered the data of the two centres that were excluded the participation rate would have been similar (36.9%).

**Table 1:** Clinician participation by centre

Centre	All invited	Number of participants	Participation %
**Hospitals (n=15)**			
Catalonia			
H. de Sant Pau	118	81	**68.6**
H. U. Germans Trias i Pujol	54	35	**64.8**
C. Sanitari de Terrassa	43	27	**62.8**
H. General de Granollers	30	17	**56.7**
H. Sant Joan Despí M. Broggi	47	20	**42.6**
Corporació Sanitaria Parc Taulí	69	28	**40.6**
H. de Sant Rafael	35	14	**40.0**
Fundació Salut Empordà	25	10	**40.0**
Consorci Sanitari de l'Anoia	25	9	**36.0**
H. Sant Joan de Deu (Esplugues)	7	2	**28.6**
H. General de Catalunya	46	12	**26.1**
Madrid			
H. Universitario Ramón y Cajal	64	22	**34.4**
H. Puerta de Hierro	67	16	**23.9**
Basque Country			
H. Universitario Cruces	94	28	**29.8**
H. Universitario Donostia^b^	285	60	**21.1**
**Primary Care Centres (n=3)**			
Catalonia			
EAP Lledoners	2	2	**100.0**
EAP Sardenya	3	2	**66.7**
Consorci Castelldefels Agents de Salut	16	4	**25.0**
**Others**^a^	-	24	**-**
**Total**	**1,030**	**413**	**40.1**

^a^Others: includes participants who did not specify their centre.

^b^Donostia Hospital invited not only clinical leaders but also the entire medical team.

The median participation rate per specialty was 41.3% (range 12.5%-78.9%) (Table 2). Nine specialties had a participation rate over 50.0% (rheumatology, dermatology, nuclear medicine, medical oncology, internal medicine, occupational medicine, geriatrics, radiology and haematology); in two specialties less than 20.0% responded (anaesthesiology and urology).

**Table 2:** Participation in the survey by medical specialty.

Medical speciality	All invited	Number of participants	Participation %
Rheumatology	19	15	**78.9**
Dermatology	22	15	**68.2**
Nuclear medicine	9	6	**66.7**
Medical oncology	20	14	**66.7**
Internal medicine	34	23	**65.7**
Occupational medicine	14	9	**64.3**
Geriatrics	16	10	**58.8**
Radiology	31	17	**53.1**
Haematology	29	15	**51.7**
Endocrinology	20	10	**50.0**
Pneumology	22	11	**50.0**
Neurology	41	21	**50.0**
Preventive medicine & public health	9	5	**50.0**
Infectious diseases	20	9	**45.0**
Physical medicine & rehabilitation	19	8	**42.1**
Psychiatry	30	13	**41.9**
Emergency medicine	41	17	**41.5**
Immunology & allergology	17	7	**41.2**
Obstetrics & gynaecology	39	17	**40.5**
Microbiology	21	8	**38.1**
Biochemistry	26	10	**37.0**
Otorhinolaryngology	39	14	**35.9**
Cardiology	34	12	**35.3**
Traumatology	29	10	**34.5**
General surgery	59	21	**34.4**
Gastroenterology	34	12	**34.3**
Vascular surgery	16	6	**31.6**
Intensive medicine	29	9	**31.0**
Paediatrics	40	12	**29.3**
Family medicine	64	21	**28.8**
Ophthalmology	32	8	**25.0**
Nephrology	22	5	**22.7**
Anaesthesiology	101	19	**18.6**
Urology	32	4	**12.5**
**Total**	**1030**	**413**	**40.1**

**Fig. 1:**
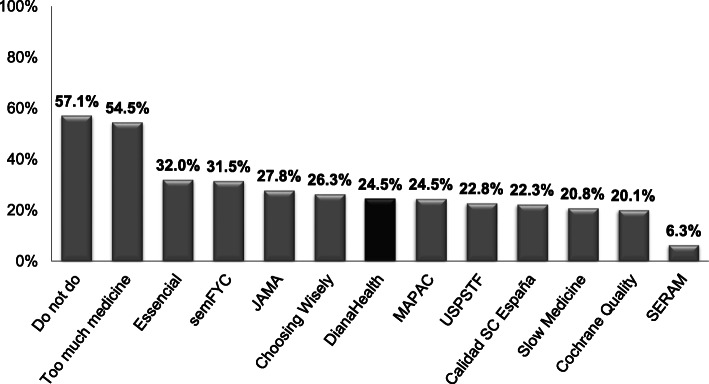
Awareness of initiatives identifying non-recommended practices included in DianaHealth® (*N* = 413).

A total of 82.6% of participants (IQR 77.9%-94.9%) reported that they knew of at least one of the 12 initiatives identifying non-recommended practices included in DianaHealth (Table 3 and Fig. 1). The mean number of known initiatives per specialty was 4 (range 1-12), the *Do Not Do* and *Too Much Medicine* initiatives being the most widely-known, with 57.1% and 54.5% or respondents, respectively, being aware of them (Fig. 1). Most initiatives were familiar to fewer than 30% of participants. Regarding national initiatives, *Essencial*, *No Hacer* (by the Spanish Society of Family and Community Medicine), and *MAPAC* were the most widely-known initiatives with awareness rates of 32.0%, 31.5%, and 24.5%, respectively.

**Table 3:** Awareness and opinion of the 12 initiatives to improve appropriateness, by medical specialty

Medical Specialty	N° of Partic.	Awareness^a^ %	N. ° of initiatives			Useful		Applicable
			Median	Range			%	%
Anaesthesiology	19	**89.5**	4	1	-	10	**100.0**	**94.7**
Gastroenterology	12	**83.3**	4	1	-	8	**75.0**	**66.7**
Biochemistry	10	**100.0**	4	1	-	10	**100.0**	**80.0**
Cardiology	12	**100.0**	5	2	-	11	**16.7**	**25.0**
General surgery	21	**80.9**	3	1	-	8	**85.7**	**76.2**
Vascular surgery	6	**100.0**	3	1	-	6	**83.3**	**83.3**
Dermatology	15	**80.0**	2	1	-	5	**80.0**	**73.3**
Endocrinology	10	**80.0**	3	2	-	9	**80.0**	**70.0**
Geriatrics	10	**90.0**	7	2	-	12	**80.0**	**100.0**
Haematology	15	**66.7**	2	1	-	6	**73.3**	**46.7**
Infectious diseases	9	**77.8**	4	2	-	8	**100.0**	**88.9**
Immunology & allergology	7	**14.3**	1	1	-		**14.3**	**42.8**
Occupational medicine	9	**66.7**	3	1	-	7	**88.9**	**77.8**
Family medicine	21	**95.2**	5	1	-	12	**100.0**	**95.2**
Physical medicine & rehabilitation	8	**87.5**	3	1	-	6	**87.5**	**62.5**
Intensive medicine	9	**88.9**	4	2	-	10	**100.0**	**88.9**
Internal medicine	23	**78.3**	6	2	-	11	**82.6**	**69.6**
Nuclear medicine	6	**100.0**	3	1	-	6	**100.0**	**100.0**
Preventive medicine & public health	5	**100.0**	7	2	-	12	**60.0**	**60.0**
Microbiology	8	**75.0**	3	1	-	10	**87.5**	**87.5**
Nephrology	5	**100.0**	3	1	-	4	**80.0**	**80.0**
Pneumology	11	**81.8**	3	1	-	5	**90.9**	**90.9**
Neurology	21	**80.9**	3	1	-	8	**57.1**	**57.1**
Obstetrics & gynaecology	17	**94.1**	4	2	-	8	**88.2**	**82.3**
Ophthalmology	8	**66.7**	2	1	-	6	**37.5**	**62.5**
Medical oncology	14	**78.6**	4	2	-	6	**28.6**	**21.4**
Otorhinolaryngology	14	**50.0**	3	1	-	7	**64.3**	**71.4**
Paediatrics	12	**75.0**	4	2	-	6	**66.7**	**75.0**
Psychiatry	13	**84.6**	5	2	-	8	**84.6**	**84.6**
Radiology	17	**94.1**	5	2	-	9	**82.3**	**82.3**
Rheumatology	15	**80.0**	3	2	-	6	**73.3**	**73.3**
Traumatology	10	**100.0**	3	1	-	5	**80.0**	**70.0**
Emergency medicine	17	**70.6**	4	1	-	7	**94.1**	**88.2**
Urology	4	**100.0**	3	1	-	7	**100.0**	**75.0**
Overall	**413**	**82.6**	4	1	-	12	**82.4**	**75.6**

^a^At least 1 initiative

Overall, perceived usefulness and applicability to clinical practice of the different initiatives was high: 82.4% (IQR 73.3%-90.4%) and 75.6% (IQR 67.4%-86.8%), respectively (Table 3). However, in four specialties (cardiology, haematology, immunology and allergy, and medical oncology) less than half of the participants considered the initiatives useful or applicable in their clinical practice.

Overall, a median of 63.1% of respondents were aware of the recommendations for their specialty (IQR 53.6%-77.5%) (Table 4). The three specialties with the highest awareness were endocrinology, anaesthesiology and radiology.

**Table 4:** Awareness, agreement, and perceived usefulness regarding recommendations, by medical specialty

	N° of Partic.	N° of Recom.	Awareness				Agreement				Usefulness			
			Median (%)	IQR (%)			Median (%)	IQR (%)			Median (%)	IQR (%)		
Anaesthesiology	19	9	**89.5**	84.2	-	100.0	**89.5**	89.5	-	94.7	**89.5**	84.2	-	89.5
Biochemistry	10	25	**60.0**	40.0	-	90.0	**90.0**	80.0	-	100.0	**90.0**	70.0	-	100.0
Cardiology	12	19	**83.3**	66.7	-	91.7	**91.7**	79.2	-	100.0	**91.7**	83.3	-	100.0
Dermatology	15	12	**40.0**	18.3	-	53.3	**76.7**	56.7	-	86.7	**66.7**	60.0	-	80.0
Emergency medicine	17	9	**52.9**	47.1	-	64.7	**88.2**	76.5	-	94.1	**82.4**	76.5	-	88.2
Endocrinology	10	23	**90.0**	80.0	-	100.0	**90.0**	70.0	-	100.0	**90.0**	80.0	-	95.0
Family medicine	21	17	**81.0**	52.4	-	90.5	**95.2**	81.0	-	95.2	**85.7**	76.2	-	95.2
Gastroenterology	12	22	**83.3**	75.0	-	91.7	**87.5**	83.3	-	100.0	**87.5**	83.3	-	100.0
General surgery	21	13	**52.4**	42.9	-	71.4	**71.4**	57.1	-	81.0	**71.4**	57.1	-	81.0
Geriatrics	10	8	**75.0**	70.0	-	82.5	**75.0**	70.0	-	85.0	**80.0**	70.0	-	90.0
Haematology	15	8	**76.7**	51.7	-	81.7	**76.7**	65.0	-	80.0	**76.7**	71.7	-	81.7
Immunology & allergology	7	18	**57.1**	57.1	-	57.1	**85.7**	71.4	-	100.0	**85.7**	71.4	-	100.0
Infectious diseases	9	13	**55.6**	33.3	-	55.6	**77.8**	77.8	-	88.9	**77.8**	66.7	-	88.9
Intensive medicine	9	10	**61.1**	55.6	-	83.3	**72.2**	0.0	-	86.1	**88.9**	88.9	-	100.0
Internal medicine	23	22	**60.9**	53.3	-	68.5	**78.3**	66.3	-	91.3	**78.3**	69.6	-	90.2
Medical oncology	14	24	**64.3**	44.6	-	78.6	**75.0**	57.1	-	85.7	**71.4**	55.4	-	85.7
Microbiology	8	24	**62.5**	46.9	-	87.5	**87.5**	71.9	-	100.0	**93.8**	71.9	-	100.0
Nephrology	5	7	**40.0**	30.0	-	60.0	**40.0**	20.0	-	60.0	**40.0**	30.0	-	60.0
Neurology	21	23	**47.6**	38.1	-	69.1	**95.2**	81.0	-	100.0	**90.5**	76.2	-	95.2
Nuclear medicine	6	13	**66.7**	66.7	-	100.0	**100.0**	83.3	-	100.0	**83.3**	83.3	-	100.0
Obstetrics & gynaecology	17	25	**70.6**	52.9	-	88.2	**88.2**	64.7	-	94.1	**88.2**	70.6	-	94.1
Occupational medicine	9	11	**77.8**	61.1	-	88.9	**88.9**	77.8	-	88.9	**88.9**	83.3	-	88.9
Ophthalmology	8	6	**43.8**	37.5	-	59.4	**81.3**	75.0	-	87.5	**75.0**	75.0	-	84.4
Otorhinolaryngology	14	8	**67.9**	57.1	-	75.0	**100.0**	94.6	-	100.0	**100.0**	92.9	-	100.0
Paediatrics	12	25	**83.3**	75.0	-	100.0	**91.7**	91.7	-	100.0	**91.7**	91.7	-	100.0
Physical medicine & rehabilitation	8	7	**62.5**	18.8	-	93.8	**0.0**	0.0	-	87.5	**75.0**	75.0	-	93.8
Pneumology	11	24	**63.6**	31.8	-	86.4	**90.9**	43.2	-	90.9	**90.9**	43.2	-	90.9
Preventive medicine & public health	5	12	**20.0**	0.0	-	30.0	**60.0**	40.0	-	65.0	**50.0**	40.0	-	60.0
Psychiatry	13	14	**61.5**	34.6	-	67.3	**73.1**	69.2	-	82.7	**76.9**	63.5	-	84.6
Radiology	17	24	**88.2**	76.5	-	94.1	**97.1**	92.7	-	100.0	**97.1**	94.1	-	100.0
Rheumatology	15	19	**46.7**	16.7	-	60.0	**80.0**	73.3	-	86.7	**73.3**	66.7	-	86.7
Traumatology	10	8	**50.0**	37.5	-	65.0	**70.0**	57.5	-	72.5	**60.0**	52.5	-	72.5
Urology	4	17	**75.0**	50.0	-	75.0	**50.0**	25.0	-	75.0	**50.0**	25.0	-	100.0
Vascular surgery	6	12	**83.3**	66.7	-	87.5	**83.3**	79.2	-	100.0	**91.7**	83.3	-	100.0
Overall	**413**	**531**	**63.1**	**53.6**	-	**77.5**	**84.5**	**75.0**	-	**90.0**	**84.5**	**75.0**	-	**90.0**

The overall median agreement with recommendations was 84.5% (IQR 75.0%-90.0%). The five specialties with the highest reported agreement were nuclear medicine otorhinolaryngology, radiology, family medicine and neurology (Table 4).

The recommendations were perceived as useful by a median of 84.5% overall (IQR 75.0%-90.0%). The specialists that considered the recommendations more useful were those of otorhinolaryngology, radiology and microbiology (Table 4).

**Fig. 2:**
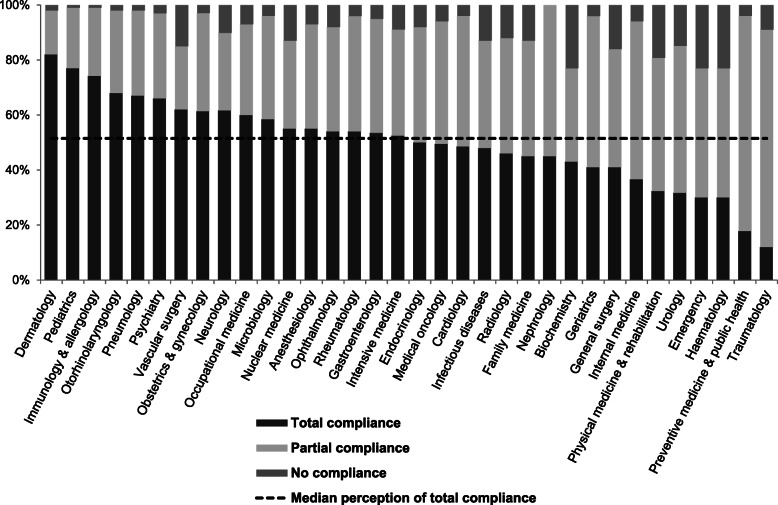
Perceived total compliance by specialty with their corresponding recommendations.

Among those specialists who agreed with the recommendations, for all specialties, a median of 51.5% (IQR 41.4%-60.9%) perceived their department as being fully-compliant, and 40.1% (IQR 31.9%-46.8%) perceived partial compliance (Fig. 2). Dermatology, paediatrics, and immunology and allergy had the highest perception of full compliance. A median 7.1% perceived their department as non-compliant with the recommendations (IQR 3.7%-12.9%).

## Discussion

This study aimed to determine clinical leaders’ awareness, agreement and interest regarding existing initiatives to reduce low-value clinical practices as well as the published recommendations specific to each specialty.

In this first approach we decided to focus our survey on recommendations on diagnostic practices and leave the development of a similar survey on therapeutic aspects for future studies.

The DianaHealth portal made obtaining the existing recommendations very easy. This open-access portal, available in English and Spanish, periodically classifies the recommendations generated from national and international initiatives aimed at reducing low-value clinical practices and improving clinical appropriateness [8].

Survey participation was not particularly high (40.1%, or 47.4% with the exclusion of the centre that invited all medical staff instead of only clinical leaders), although this is in line with results from other similar surveys. The wide variability of participation across the different centres could reflect the presence or absence of active institutional policies to reduce low-value practices. The participation rate for the different specialties was probably influenced to some extent by the specific characteristics of each centre.

Most participants claimed to know of at least one of the initiatives, although on average 4 of the 12 initiatives were known. The only initiatives familiar to at least half of the participants were *Do Not Do* and *Too Much Medicine*. The Spanish initiatives (*Essencial, No Hacer,* and *Compromiso por la Calidad*) were known to less than one third of the participants. Three-quarters of the participants judged the above-mentioned initiatives positively, both in terms of usefulness and applicability, although this perception was much lower in four specialties. Additional research about this difference should be conducted in future studies.

Around 60% of the participants reported that they knew the diagnostic recommendations for their specialty, with substantial variability between specialties. Three-quarters of the participants agreed with the recommendations and considered them useful. It would be interesting for future studies to analyse in depth the differences between specialties regarding perceived usefulness. Around 50% of the participants estimated that professionals at their centre always complied with the recommendations, 40% estimated partial compliance, and less than 10% estimated non-compliance, with some variability between specialties.

Despite the many initiatives and projects generating recommendations to reduce low-value clinical practices, very few studies have been conducted to assess target physicians’ perception of these recommendations. Our survey is the first to provide an overall assessment of existing local, national and global initiatives, by evaluating awareness and perceived usefulness. DianaHealth has proven to be a very valuable resource for identifying potential questionable practices.

One of the potential limitations of our study is that participation was lower than desired. Nevertheless, our response rate was in relation to has been described for other web surveys [25, 26]. Most of the participating centres were in Catalonia, because the survey coordinators were based there, and also because most of such initiatives have been developed there. These local initiatives are generally promoted in hospitals, which is why fewer primary care professionals were invited to participate. Another limitation of our study is a potential self-selection bias because of participants who answered the survey could be those more aware and familiar with the importance of preventing low-value care.

The response rate was quite good (40%) in relation to what is described for web surveys [25, 26]. The research group followed the methodological steps of this type of survey to improve the response: make a pilot of the survey (to assess the design, order and duration), make the invitation to participate, send a reminder and ensure easy access to the questionnaire [25]. However, no incentive was offered.

## Conclusions

Overall, there is insufficient awareness among Spanish clinical leaders regarding worldwide initiatives to improve health care appropriateness, despite perceived usefulness being high. Furthermore, according to the participants, diagnostic recommendations specific to each specialty are widely known, agreed with and adhered to at each centre, which is very encouraging. It seems necessary for such initiatives, which periodically generate recommendations on reducing low-value practices, to develop additional knowledge-translation actions addressed at the target physicians to improve awareness and compliance.

## Data Availability

The datasets generated and analysed during the current study are included in the Clinapsis® electronic platform repository, but restrictions apply to the availability of these data, and they are not publicly available. Data are however available from the authors upon reasonable request.

## Abbreviations

IQR: Interquartile range
JAMA: Journal of the American medical association
NICE: National institute for health and care excellence
MAPAC: In Spanish*, “Mejora de la Adecuación de la Práctica Asistencial y Clínica”*; in English, Improvement of Appropriateness in Clinical Practice and Healthcare
